# Bioprinting of Perfusable, Biocompatible Vessel-like Channels with dECM-Based Bioinks and Living Cells

**DOI:** 10.3390/bioengineering11050439

**Published:** 2024-04-29

**Authors:** Marta Klak, Michał Rachalewski, Anna Filip, Tomasz Dobrzański, Andrzej Berman, Michał Wszoła

**Affiliations:** 1 Foundation of Research and Science Development, 01-242 Warsaw, Poland or michal.wszola@polbionica.com (M.W.); 2 Polbionica sp. z o.o., 01-242 Warsaw, Poland

**Keywords:** bioprinting, vessels, endothelial cells, bioink, perfusion, extracellular matrix

## Abstract

There is a growing interest in the production of bioinks that on the one hand, are biocompatible and, on the other hand, have mechanical properties that allow for the production of stable constructs that can survive for a long time after transplantation. While the selection of the right material is crucial for bioprinting, there is another equally important issue that is currently being extensively researched—the incorporation of the vascular system into the fabricated scaffolds. Therefore, in the following manuscript, we present the results of research on bioink with unique physico-chemical and biological properties. In this article, two methods of seeding cells were tested using bioink B and seeding after bioprinting the whole model. After 2, 5, 8, or 24 h of incubation, the flow medium was used in the tested systems. At the end of the experimental trial, for each time variant, the canals were stored in formaldehyde, and immunohistochemical staining was performed to examine the presence of cells on the canal walls and roof. Cells adhered to both ways of fiber arrangement; however, a parallel bioprint with the 5 h incubation and the intermediate plating of cells resulted in better adhesion efficiency. For this test variant, the percentage of cells that adhered was at least 20% higher than in the other analyzed variants. In addition, it was for this variant that the lowest percentage of viable cells was found that were washed out of the tested model. Importantly, hematoxylin and eosin staining showed that after 8 days of culture, the cells were evenly distributed throughout the canal roof. Our study clearly shows that neovascularization-promoting cells effectively adhere to ECM-based pancreatic bioink. Summarizing the presented results, it was demonstrated that the proposed bioink compositions can be used for bioprinting bionic organs with a vascular system formed by endothelial cells and fibroblasts.

## 1. Introduction

Biomedical engineering is a fast-developing discipline of science that combines the achievements of engineering and life sciences towards the restoration and fabrication of complex physiological systems [1,2]. Despite the huge potential of the technology, there are still numerous problems to be solved. The choice of appropriate materials and the lack of stable, perfused vasculature are two of the many factors involved [3,4,5]. The major challenge for biofabrication is mimicking the natural extracellular matrix (ECM) due to its unique composition of molecules, which cannot be underestimated [1,6]. Bioinks based on tissue extracellular matrix (ECM) are gaining appeal as a solution to this challenge because they allow structures containing living cells to retain biological activity. The preparation of ECM-based bioink proceeds with the decellularization of native tissue to remove cells and tissue debris, thereby removing antigens, which could cause an immune or inflammatory response [7]. In parallel, the process must be conducted with caution to preserve the ultrastructure and composition of the ECM [8,9]. Human-derived ECM is most desirable for clinical applicability; however, current studies on it are scarce and ethically questionable [10]. Nevertheless, promising, pragmatic, and reasonable alternatives are xenogeneic sources of the ECM, which are obtained from diverse tissue sources of varied animal species; however, porcine tissues are considered a major source [1,11,12]. The most spectacular and convincing hydrogel usage of a porcine-derived ECM as clinically applicable is the case of the first-in-men trial, where injection in early and late post-myocardial infarction patients with left ventricular dysfunction were performed [13]. Xenogeneic dECM-based (decellularized extracellular matrix) hydrogels were successfully adapted in numerous studies [1,10,14,15,16,17]. Nevertheless, dECM itself is a single component of bioinks, which predominantly are a blend of various materials. Therefore, a suitable bioink, which would be a reasonable trade-off between biocompatibility and mechanical performance, is highly needed. Recently, gelatin methacrylate (GelMa) seems to be in the spotlight. Although viscosity and shear-thinning properties do not allow for the use of GelMa in its basic form for direct printing [18], it is becoming more attractive as an additive to hydrogels due to the favorable biological properties of gelatin and photo-crosslinkable behavior caused by methacrylamide groups [10,19,20,21]. To date, GelMa was successfully used in cardiac, cartilage, vascular, and skeletal muscle tissue engineering [22]. In all studied cases, modifications of bioinks containing GelMa were needed to enhance and enable the printability of crafted bioink. Thus, hyaluronic acid, collagen I, gellan gum, nanosilicates, and methylcellulose are often incorporated to increase rheological benefits and improve printability [23,24,25,26,27,28,29]. Finally, another crucial group of components for bioink fabrication are photoinitiators, which enhance the polymerization of biomaterials. Light exposure, predominantly UV, is a common form of post-printing polymerization (photopolymerization) because it is environmentally friendly, cost-effective, and extremely fast [30]. However, cell survival is important and should be considered during the bioprinting process. Thus, crosslinking process with visible light of 405 nm wavelength is preferable since it induces minimal DNA lesions to cells when compared with the UV-A crosslinking method [31]. Undoubtedly, lithium phenyl-2,4,6-trimethylbenzoylphosphinate (LAP) is one of the most frequently used due to its polymerization rates in visible light [32,33].

Although choosing appropriate materials is essential for bioprinting, there is, however, another, equally important issue related to the inclusion of a vasculature system within fabricated scaffolds. A vasculature system is essential for any scaffold that in diameter exceeds 200 µm, as this value constitutes a limit for oxygen diffusion [34]. Moreover, blood vessels are responsible for delivering nutrients and vital components but also, simultaneously removing waste products [35,36]. Thus, different methods were tested to enhance the infiltration rate of blood vessels in newly formed scaffolds. Presently, it is estimated to be too low (<1 mm/day) to be used in clinically sized organs [4,37]. Hence, the in vivo implantation of large scaffolds generated in vitro is doomed to fail if the vascularization of scaffolds is not achieved [38]. The viability of cells embedded in the implanted scaffold is compromised in a case when it is more than 150–200 nm away from functioning vasculature [39]. The promotion of the vascularization of tissue engineered constructs is performed with the appropriate design of scaffolds with adequate porosity, the delivery of angiogenic growth factors, and the surface immobilization of peptides that promote angiogenesis [40]. In the process of vascularization, many cell types and co-cultures are used depending on the approach that the researcher has adopted. Thus, for the vascularization process, endothelium cells, fibroblasts, smooth muscle cells, and stem cells are often used in different pairwise combinations [41,42,43]. Hence, the role of ECs is related to the generation of extracellular vesicles, which are cellular components responsible for intracellular communication necessary for crosstalk with the environment and capable of transferring substances that influence the behavior of other cells. Additionally, ECs generate specific paracrine factors fundamental for forming cell–cell interactions. Supporting cells, specifically, fibroblasts, are obligatory, but apparently, their activity is restricted only to the initial phase of vasculogenesis, and they could be ablated without affecting previously established vasculature [44]. Nevertheless, specific genes expressed by fibroblasts were found to be crucial for lumenogenesis, which does not occur in single-cultured ECs [45]. Although the function of fibroblasts is origin dependent, it is considered that their primary role is related to the creation of a functional vascular system.

Alternatively, methods for fabricating perfusable channels with adjustable dimensions and forms utilizing diverse technologies were explored as an alternative to the de novo vessel construction process [46,47,48]. Thus, embedded microchannels within hydrogels were one of the strategies where various sacrificial materials were incorporated, including Pluronic, GelMa, gelatin, and alginate [49,50,51]. Byambaa et al. used a technique of printing a GelMa fiber that is highly degradable and, when surrounded by other materials, forms a perfusable vessel [52]. Bioprinting with coaxial needles, however, refers to the extrusion of two separate fibers composed of different materials where the outer layer forms a shell, whereas the inner layer is an easily removable core responsible for the stabilization of an outer layer [53,54]. Despite numerous studies, there is a need for the development of an appropriately designed, extrusion-based bioprinting model of tunable-in-size vessels, which could be a platform for further improvement. However, despite many attempts, no effective method of producing a vascular system has been demonstrated so far. One of the problems is focusing only on the type of biomaterial used to create the blood vessels. Such an approach cannot but bring satisfactory results. This was also demonstrated by the team of Galvan et al., who, in their in vivo studies on a pig model, used a model made of PEGDA with the addition of heparin as an antithrombotic agent. Despite such a composition of the bioink, the Doppler-controlled flow was detectable for only 5 h [55,56,57].

Lee et al. [56] also attempted to create a vascular system. To this end, they developed a model that allows for perfusion through a 3D printed layer-by-layer model. In their study, Lee’s team used gelatin and collagen from rat tails, which were mixed with HUVEC cells. Lee’s research was among the first to use research with natural biocomponents. In earlier scientific papers, you can read about an attempt to produce a vascular system using microfabrication technology [56]. However, these studies used artificial components, such as PDMS or polymers, in microfluidic systems that prevent internal tissue remodeling. Comparing the available literature data, we concluded that technology using 3D bioprinting with living cells can open new doors for tissue engineering and regenerative medicine. However, to make this possible, it is necessary to optimize the bioprinting technology in terms of technology, biomaterials, and cell concentration.

The study aimed to optimize the production of the perfusable vessels-like channels of the bioink consisting of a decellularized extracellular matrix and methacrylic substances with living endothelial and fibroblast cells using a 3D-bioprinting extrusion machine. The composition of the proposed bioink enables the printing of a three-dimensional and stable flow system. Additionally, the proposed bioink is a biocompatible material, and thus, endothelial cells and fibroblasts willingly inhabit its inner surface. To demonstrate these properties, experiments were performed with red fluorescent human skin fibroblasts (HDFa) and green fluorescent human umbilical vein endothelial cells (HUVEC). Two spatial arrangements were tested to investigate the most appropriate way to inject cells for optimal adhesion.

## 2. Materials and Methods

### 2.1. Cells

We used red fluorescent human dermal fibroblasts (HDFa) and green fluorescent human umbilical vein endothelial cells (HUVEC) as a model. HDFa cells from ATCC were grown in Fibroblast Growth Kit-Low-Serum (ATCC, VA, USA, cat no.: ATCC^®^ PCS-201-041) medium according to manufacturer instructions. Endothelial cells were delivered from InnoProt as a TTFLUOR™HUVEC cell and grown in manufacturer media (Bizkaia, Spain; cat.no.: P60104). Those two types of cells were selected due to their application in biocompatibility and angiogenic research and efficient proliferation while co-cultured [44,56,57,58,59,60].

### 2.2. Bioink Preparation

The material for decellularization was obtained from a local slaughterhouse, and immediately after dissection, the pancreatic tissue was thoroughly cleared of fat, large vessels, and connective tissue and stored in PBS (phosphate-buffered saline) (Tablets, Takara; Saint-Germain-en-Laye, France; cat. no.: T9181)/streptomycin (Merck; St. Louis, MO, USA) solution before further proceeding. The whole process was conducted according to the protocol published by Klak et al., 2021 [61].

#### 2.2.1. Bioink A for 3D Channel Bioprinting

Bioink A consisted of 3 components: 5% dECM-based hydrogel and methactyl substances: 20% methacrylated gelatin (GelMa) and 2% methacrylated hyaluronic acid (HAMA).

To prepare a 5% (*w*/*v*) hydrogel with dECM, pepsin (Pepsin powder BRP, European Pharmacopoeia Reference Standard, Strasbourg, France; cat. no.: P0525000) was weighed into a 0.01 M HCl solution and dissolved according to the manufacturer’s instructions. The dECM powder was weighed into the prepared solution. The final concentration of the dECM solution was 10%. The hydrogel was left on a magnetic stirrer for 72 h at a mixing speed of 500 rpm in a water bath (30 °C). After this time, the neutralization process began so that the final pH of the solution was 7.37. After neutralization, the solution was stored in the refrigerator.

To prepare the GelMa (Polbionica Sp. z o.o., Warszawa, Poland, cat. no.: TNT02) solution and the 1×PBS solution, freeze-dried methacrylated gelatin was weighed, and then, the LAP photoinitiator (Lithium phenyl-2,4,6-trimethyl-benzoyl phosphinate) was added at a final concentration of 0.25% (*w*/*v*). The GelMa solution with the photoinitiator was left in a water bath (50 °C) until completely dissolved, and the mixing speed was selected from the degree of foaming to 100–200 rpm. After GelMa was fully dissolved, the pH of the solution was checked, and it was 7.17. To adjust the pH, 5 µL of 5M NaOH was added, and the pH value was 7.33. The prepared solution was filtered using a 0.22 µm syringe filter. The filtered solution was transferred to a 50 mL falcon wrapped in aluminum foil and placed in the refrigerator.

To prepare the HAMA (Polbionica Sp. z o.o., Warszawa, Poland, cat. no.: TNT03) solution, 1×PBS solution was used, to which LAP (final concentration 0.5%) and lyophilized hyaluronic acid (final concentration 2%) were weighed. The mixture prepared in this way was transferred to a thermoblock and shaken at a speed of 1000 rpm and a temperature of 8 °C for 1 h. The final pH of the solutions was 4.12 and 4.01, respectively. An amount of 5 M NaOH was added to adjust the pH, and the final pH was 7.44. Then, the solutions were filtered using 0.22 µm syringe filters. The filtered solutions were sterilely transferred to 50 mL falcons wrapped in aluminum foil and stored in a refrigerator.

Before the bioprinting process, crosslinking agents were added to the dECM-based hydrogel. GelMa and HAMA were added in proportions of 2:3, and the mixture of methacrylic substances for dECM hydrogels was mixed in 1:1 proportion. The blend was applied for extrusion bioprinting. During the bioprinting process, the material was crosslinked using 405 nm wavelength light [31].

#### 2.2.2. Cell-Laden Bioink B Used to Seed Cells in 3D Bioprinting Process

To prepare bioink B, an appropriate amount of dECM was suspended in the PBS×1 solution. The falcon with the suspension was placed in an ice bath, taking care not to shake the suspension. Then, the hydrogel sonication process began. A sonicator, Bandelin Sonopuls HD 3100 (Bandelin electronic GmbH & Co. KG, Berlin, Germany), and a TS 103 sonotrode were used, the amplitude was set to 60%, the sonication time was 5 min, with the process interrupted every 30 s to wait until the temperature dropped to 22 °C. The prepared hydrogel was tightly closed and placed in the refrigerator. Before use in further stages, the falcon with hydrogel was heated in a thermoblock at 37 °C with stirring at 800 rpm; this was to liquefy and de-aerate the hydrogel.

### 2.3. Experimental Setup

Bioprinted scaffolds presented in this study were performed with BIO X^TM^ 3D bio-printer (CELLINK, Gothenburg, Sweden). They were printed using 609 μm nozzle, and in every case, the print bed temperature and a part of the bioprinter where construct was manufactured was set to 19 °C. Due to different printing conditions for bioink A and B, two separate printhead mouths were used. Details regarding temperature and pressure while bioprinting are presented in sections where the description of the following constructs appears.

#### 2.3.1. Experimental Chamber

**Figure 1:**
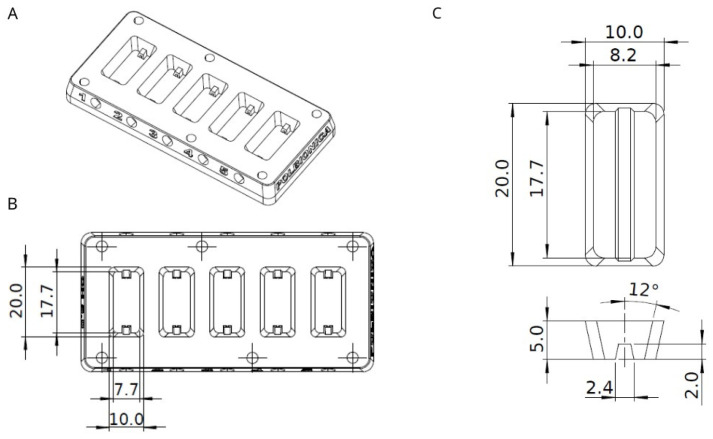
(**A**) Sketch of experimental chamber; (**B**) Experimental chamber divided to five equal-sized compartments with detailed dimensions; (**C**) Shape and dimensions of printed vessel fabricated of hydrogel A. All measurements are in millimeters.

A closable experimental chamber was designed in Solidworks software (Figure 1), which was adjusted before 3D printing with Formlabs PreForm software. The chamber was manufactured by Polbionica Ltd., made of biomedical clear resin (Form Labs, Somerville, MA, USA), and composed of five independent compartments (Figure 1) where vessels made of hydrogel A were printed. The bottom side of a chamber was made of slide glass to observe vessel content. The opposite sides of each compartment were equipped with luer lock connectors, which allowed for it to set the media to flow through the construction in a closed system.

#### 2.3.2. Spatial Arrangement of Bioink A Fiber

**Figure 2:**
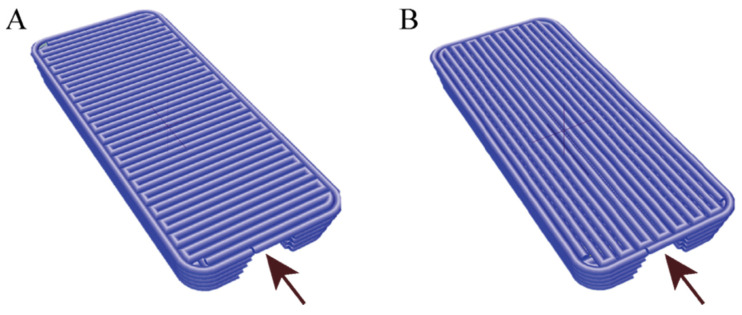
Model of vessel construction with vault composed of fiber printed perpendicularly (**A**) or parallelly (**B**) to direction of media flow. Arrowheads indicate direction of media flow through 3D printed vessel.

Two types of g-code files were designed (Figure 2). The two types of files were intended to demonstrate whether the positioning of individual fibers during the bioprinting process affects cell adhesion. The top layers of a channel, which form a vault and simultaneously have a contact with media, were printed either perpendicularly or parallelly to the direction of a media flow. Bottom side of a vessel was attached to side glass, which allowed for the observation of cells inside it. After 3 and 10 days of incubation with flow, channels proceeded with immunohistochemistry staining and cut into orientation that gave “U”-shaped sections. Vessels of bioink A were manufactured with the nozzle settled in a printhead mount set to the temperature of 24.3 °C. The pressure in the nozzle varied from 24 to 37 kPa, and the speed of printing a single fiber was estimated to 20 mm/s. After each layer, the printer head with the module of 405 nm UV–vis light was used for photo-crosslinking.

##### Bioprinting the Vascular System with Bioink B with Endothelial and Fibroblasts Cells—In the Bioprinting Process

**Figure 3:**
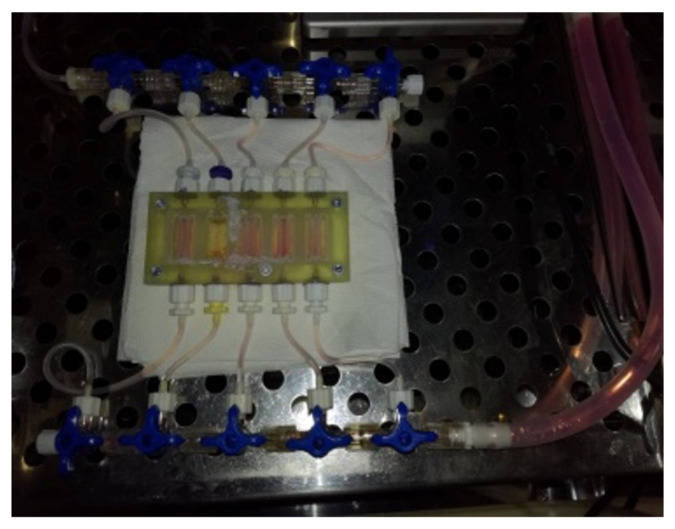
The photo shows the layout after the models are printed. Each model has a separate inlet and outlet for the flow of the culture medium. This solution allows for the simultaneous testing of 5 models in the same breeding conditions. After printing, the cartridge showed tight flow through the chamber.

The channels made of bioink A were filled with printed cell-laden bioink B of 8 million/mL cell concentration (HUVEC, HDF 1:2) and left for incubation at 37 °C with CO_2_ concentration of 5%. Applied temperature of incubation influenced the structure of hydrogel B, which became a liquid. The total volume of cell-laden hydrogel B was 50 µL per channel, which corresponded with cell quantity of 400,000. Afterward, we ran the media flow of 5 mL/min though the channel for 5 min. Cells rinsed out of the channel were counted. Bioink B used to fill the channel was printed in lower thermal regime—20 °C, lower pressure—20 kPa and decreased speed of fiber production—7 mm/s. In another variant, the incubation time was prolonged and established at 2 h, 5 h, 8 h, and 24 h. After given time points, media flow in a close system was set at 0.2 mL/min. The experiment was performed in three replicates for each time variant. The flow culture system is shown in Figure 3.

##### Seeding the Flow Channel with Cell Lines (HUVEC and HDF)—After Bioprinting Process

Prior to seeding, channels of bioink A were printed. Each channel was filled with bioprinted fiber of Pluronic in a volume of 50 µL to stiffen the construction and prevent the vault from subsiding. Such prepared chamber was placed on ice for 5 min, which resulted in liquefaction of Pluronic. Then, a one-way flow of 5 mL 1×PBS with 1 mL/min was set to wash out Pluronic. Afterwards, cells at a concentration of 8 million/mL (HUVEC, aHDF 1:2) suspended in medium were plated directly into the printed channel using an automatic pipette. Each channel was filled in this way with a volume of 50 µL cells suspended in medium. Thus, 400,000 cells were seeded in every channel. Experimental chamber was flipped upside down so the vault of a U-shaped vessel was directed down and placed in incubator at 37 °C with CO_2_ concentration of 5%. Then, flushing of a channel and cell counting was performed as described in previous experimental procedure. In another variant, the incubation time was prolonged and established at 2 h, 5 h, 8 h, and 24 h. After given time points, media flow was set at 0.2 mL/min. Three replicates were performed for each time variant.

### 2.4. 3D Channel Staining and Imaging

Three-dimensional bioprinted vessels on the 10th day of experiment were collected and fixed for 48 h in 4% (*v*/*v*) paraformaldehyde in PBS. Then, paraffin wax-embedded tissue blocks were cut into a 5 μm thick section and placed on microscope glass. Then, the standard deparaffinization procedure was carried out. Briefly, sections were deparaffinized in xylene for 5 min and rehydrated by immersing the slides in several ethanol dilutions (95%, 85%, 70%, and 50%) for 2 min each. Then, slides were rinsed in distilled water. Prepared slides were stained with hematoxylin–eosin (H&E) stain for general histological examination, and anti-ve-cadherin and anti-CD31/PECAM-1 primary antibody for the protein expression studies were used. For H&E staining, after nuclei stain with the Mayer’s hematoxylin solution for one minute, slides were washed in the tap water and incubated in eosin solution for two minutes. Then slides were washed in tap water and mounted. Immunohistochemical staining was performed using ImmPRESS Duet Double Staining Polymer Kit (Vector, Newark, CA, USA; cat. no.: MP-7724) according to the manufacturer’s instruction.

Briefly, for e-cadherin or CD31/PECAM-1 protein staining, following the standard microwave heat-induced unmasking procedure in citrate buffer (10 mM citric acid, 0.05% Tween 20, pH 6.0), slices were incubated in BLOXALL Blocking Solution (Vector, ImmPRESS Duet, MP-7724) for 10 min. After blocking in 10% normal horse serum for 1 h at room temperature, the sections were incubated with e-cadherin (Santa Cruz, Dallas, TX, USA, cat. no.: sc-8426) or CD31/PECAM-1 (Novus Biological, Centennial, CO, USA; cat. no.:NB100-2284) primary antibody overnight at 4 °C. The section used as the negative control was incubated with 10% normal horse serum overnight at 4 °C. Then, sections were washed twice for 15 min in TBST buffer and covered by ImmPRESS Duet Reagent (HRP-conjugated secondary mouse antibody and AP-conjugated secondary rabbit antibody) for 10 min at room temperature. Following the three washings in TBST buffer for 10 min each, the DAB or AP substrate (Vector, ImmPRESS Duet, MP-7724) was added to the slides. After 2–5 min of incubation, when staining was well developed, slides were washed in TBST and mounted. Imaging was performed under an Olympus X83 microscope with magnifications 20- and 40-fold using CellSene Dimension software (OLYMPUS cellSence Dimension 1.17).

### 2.5. Statistical Analysis

The significant difference from the respective controls for each experimental test condition was assessed with one-way analysis of variance (ANOVA) and Dunnett test. The difference was significant if the *p*-value was less than 0.05. Statistical analysis was performed using GraphPad Prism V5.01 software (GraphPad Software Inc., La Jolla, CA, USA).

## 3. Results

### 3.1. Evaluation of the Tested Methods of Seeding Cells into the Flow System

**Figure 4:**
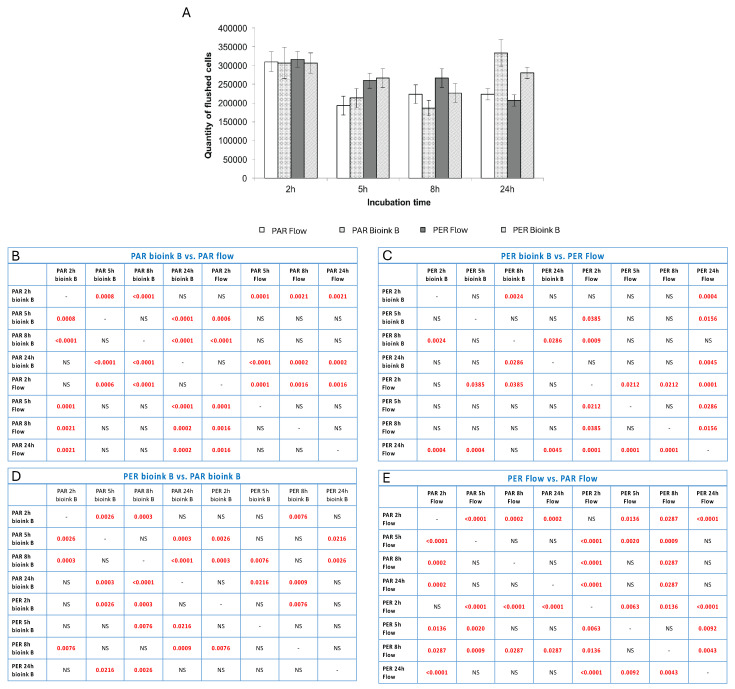
The quantity of cells flushed out from channels seeded with cells seeded in or after the bioprinting process or in given time variants of incubation. The method of fiber arrangement while printing is marked as PER (perpendicular to media flow) or PAR (parallel to media flow). The graph (**A**) shows the number of cells flushed out of the system after connecting the flow together with standard deviations. Tables (**B**–**E**) present the statistical analysis between individual experimental variants. Statistically significant values are marked in red (these are values for *p* < 0.05; NS—no statistical significance).

**Figure 5:**
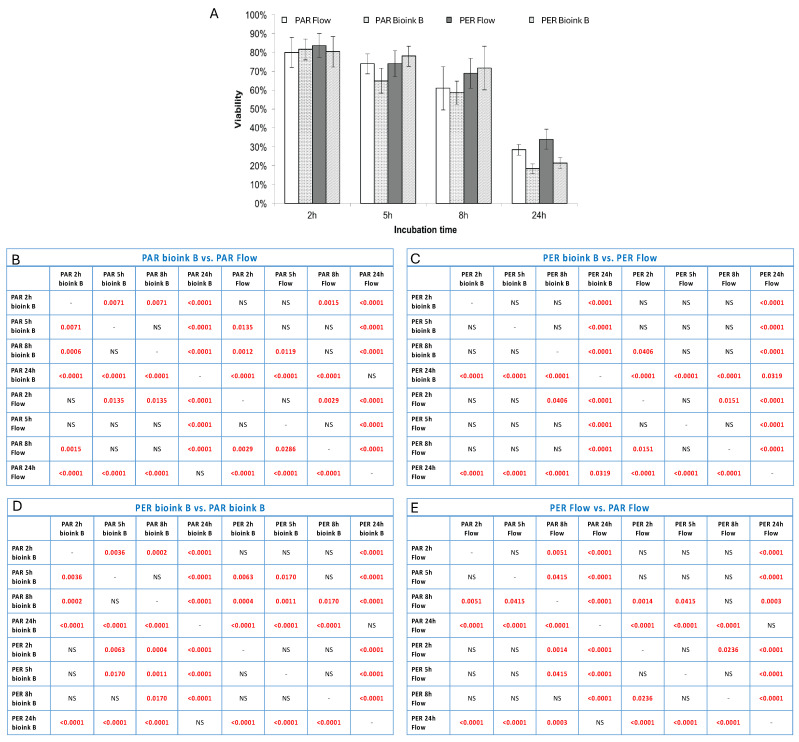
The viability of cells flushed out in tested variants of fiber arrangement and incubation time. The graph (**A**) shows the viability of cells washed out of the system after connecting the flow together with standard deviations. Tables (**B**–**E**) present the statistical analysis between individual experimental variants. Statistically significant values are marked in red (these are values for *p* < 0.05; NS—no statistical significance).

The analysis of the number of cells and their viability after selected time points showed that both the method of settling the vascular system and the technology in which it is printed are important for its final structure and stability. Based on the obtained results, it was shown that the incubation time of 2 h (in a closed system) shows the smallest discrepancies between the tested systems. This may be because 2 h is too short for endothelial cells and fibroblasts to adhere strongly enough to the biomaterial (Figure 4). It is also worth noting that at this time point (2 h of incubation), the most cells were washed away, and their viability was high (above 80%; Figure 5). In the case of longer incubation times (5 and 8 h—in a closed system), it was shown that the amount of washed-out cells was lower in systems where the channel opening was printed in a parallel manner. The number of cells washed out by the flow was on average 18–23% lower than in the case of channels printed perpendicularly to the flow lumen (Table 1, Figure 4).

**Figure 6:**
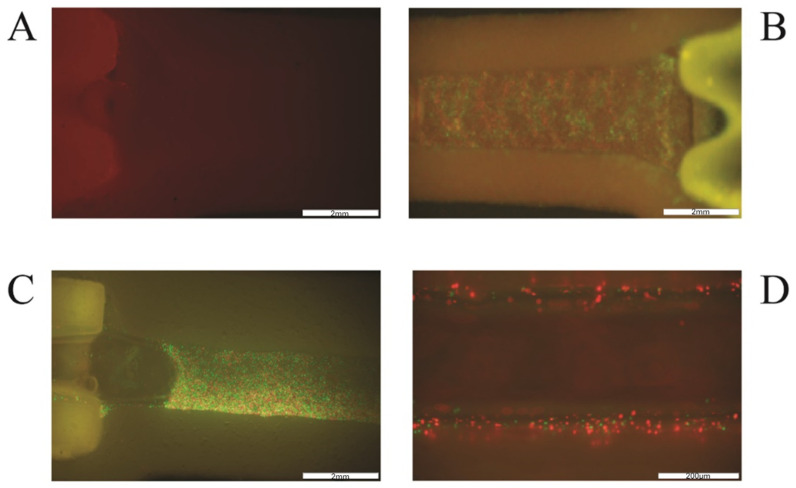
Extrusion-based printing of 3D vessel with HUVEC (green) and HDF (red) cells. (**A**): empty channel-shaped construct of hydrogel A; (**B**): channel filled with cell-laden bioink B; (**C**): channel filled with cells seeded after 3D bioprinting, after Pluronic was flushed out from the channel (**D**): channel walls with attached cells after rinsing the construct with medium. The scale in photos (**A**–**C**) is 2 mm and in photo (**D**) it is 200 μm.

**Figure 7:**
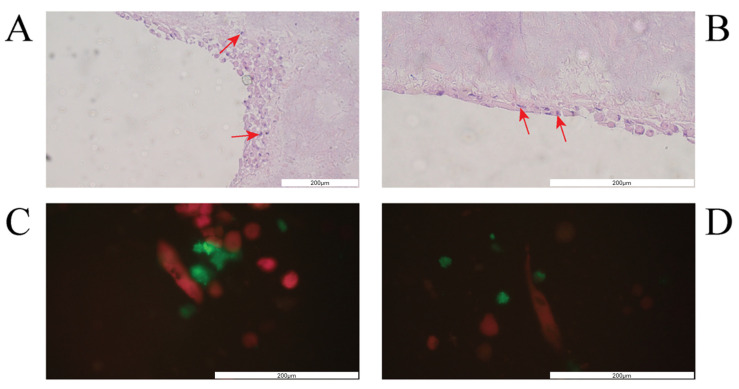
(**A**,**B**) Cells attached to channel walls and vault after 3 days of incubation with constant media flow (H&E staining). Red arrowheads indicate purple nuclei stained with hematoxylin. (**C**,**D**) Picture of HUVEC (green) and HDF (red) cells in channel with media flow after 10 days of incubation. The scale in all photos is 200 μm.

In the case of incubation for 24 h, the smallest number of leached cells was also characterized by the system using the parallel printing technique (PAR). Microscopic observations confirmed the data obtained in the cell counting stage, and the degree of adhesion to the internal walls of the canal was presented on microscopic images and H&E staining (Figure 6 and Figure 7). Cells in this type of model showed tight adherence both to the biomaterial forming the vessel lumen and to each other. Thanks to this, a monolayer of cells was created along the entire canal. Additionally, cells can be observed growing into deeper vascular structures. In the microscopic imaging of cells, both types of cells can be observed, which proves the correct selection of the cell medium, and the appropriate proportions of cells used for the bioprinting process.

Therefore, further analyses focused primarily on bioprinting of the vascular system in such a way that the lumen of the vessel was printed parallel to the direction of the flow.

A further analysis of the results in terms of the method of homing (a) in the 3D bioprinting process, i.e., the bioprinting of the vascular system with cells suspended in dECM-based bioink, and (b) manually using pipettes after printing the model showed the advantage of the first method. Bioprinting the canal with a specially developed biorus allowed for a much higher adhesion of cells to the lumen of the vessel (incubation time 5 and 8 h) while maintaining the lowest viability among cells that did not adhere. Thus, it was shown that bioprinting does not expose cells to stress, instead allowing them to maintain their ability to adhere and gradually colonize the vascular system, which can be seen in the results of immunohistological staining later in the manuscript.

Summarizing, it can be unequivocally stated that incubation for 2 h showed the lowest degree of cell adhesion to the lumen of the vessel. It is worth noting, however, that the 24 h variant also did not show satisfactory results. Although the cells showed a high percentage of adhesion, their viability was the lowest compared to the other incubation times analyzed. This situation could be caused by a small amount of cell medium per number of cells used to populate the vascular system. Therefore, the obtained results showed that the most optimal conditions are the incubation of cells colonizing the vasculature for 5 h. This experimental system demonstrated the best cell survival (over 70%) and at least 50% adhesion. The 8 h variant was also satisfactory in terms of adhesion (approx. 50%), while cell viability was lower compared to the 5 h variant. In addition, it has been shown that cells suspended in bioink and subjected to the biodication process show a higher degree of adhesion and viability than those administered manually to a previously printed construct.

The analysis of the tested variants allowed us to demonstrate that the printing method, incubation time, and type of bioink used to create the vascular system are important for its proper functioning, and equally important, it allows us to optimize the time of creating the vascular system, which is an integral part of larger constructs for which it is one route of substance delivery, nutrients, and gas exchange.

### 3.2. Morphhology and Immonohistochemistry of Bioprinted 3D Channel

**Figure 8:**
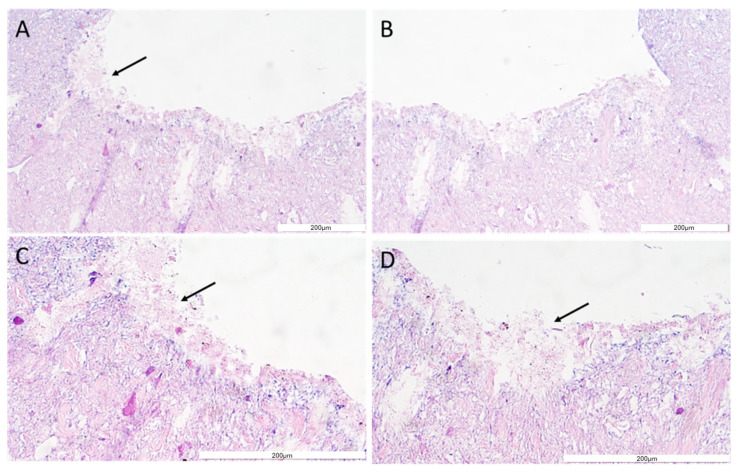
The vessel morphology. The representative pictures of hematoxylin–eosin staining are shown. The representative micrographs of the construct staining are presented at a magnification of 20-fold (**A**,**B**) and 40-fold (**C**,**D**). The micrographs were taken using Olympus microscopy. The scale bars represent 200 μm.

The structure and general morphology of the bioprinted construct as well as the ECM heterogeneity and integrity of transverse sections were studied with hematoxylin–eosin dye (Figure 8). As expected, the structure of the construct was stable, did not delaminate, and did not show excessive water absorption. The canal structure showed no leaks. The material was protected for histological imaging, and it was possible to observe cells adjacent to the vessel lumen (marked with an arrow, Figure 8). The H&E staining analysis result showed cells on the inner side of the vasculature, confirming cell adhesion to the vessel lumen.

**Figure 9:**
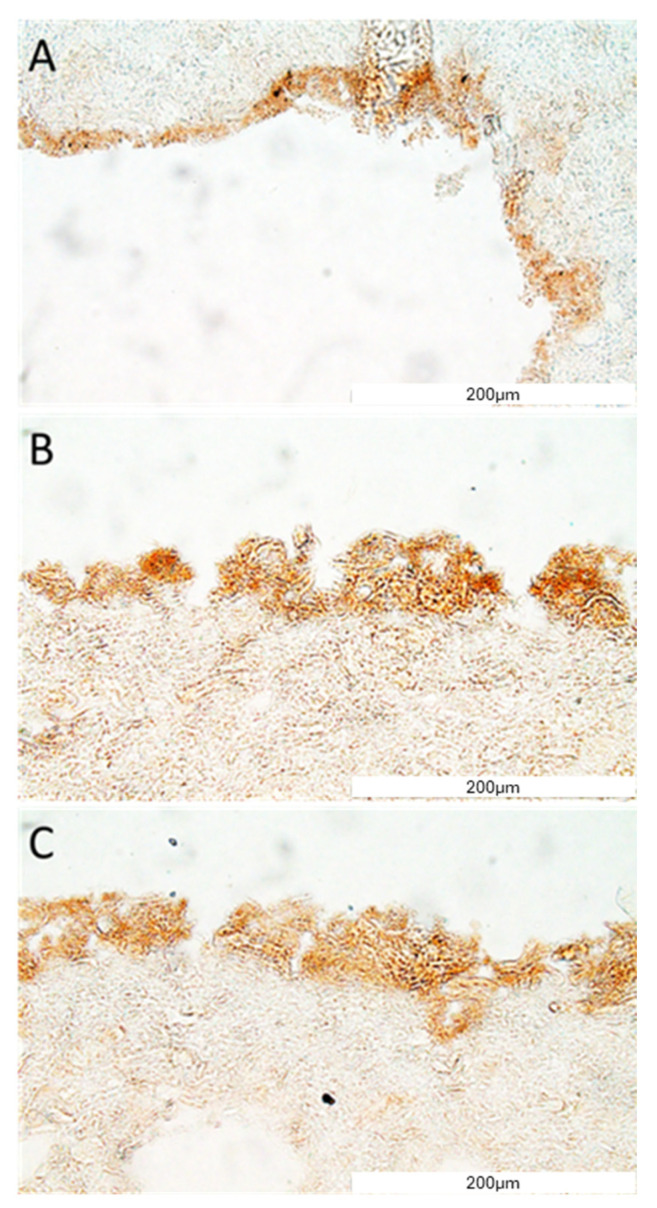
VE-cadherin protein detection in the construct section. The representative picture of the e-cadherin staining of the construct covered with cells (**A**–**C**). The micrographs were taken using Olympus microscopy at a magnification of 40×, and the scale bars represent 200 μm.

**Figure 10:**
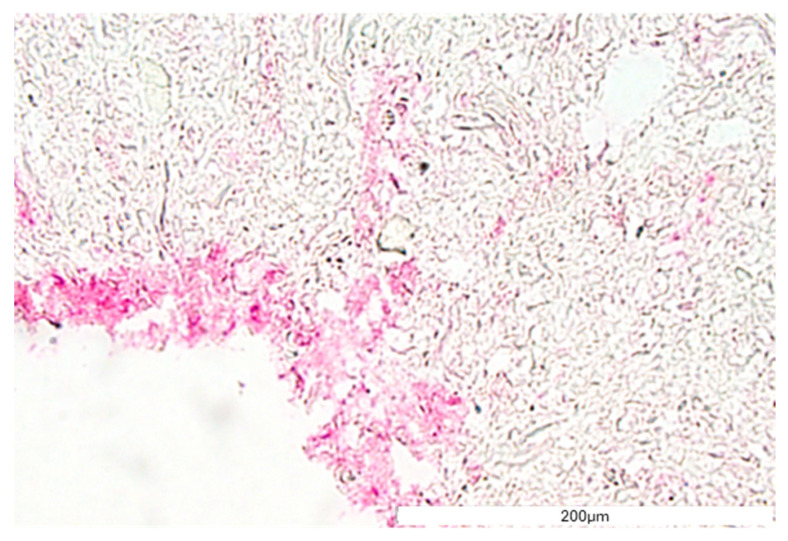
CD31/PECAM-1 protein detection in the construct section. The representative picture of the e-cadherin staining of the construct covered with cells. The micrographs were taken using Olympus microscopy at a magnification of 40×, and the scale bars represent 200 μm.

Then, the expression of e-cadherin and CD31/PECAM was revealed using immunohistochemistry. The e-cadherin as well as CD31/PECAM staining have confirmed that cells (HUVEC) were present on the surface of the construct’s channel (Figure 9 and Figure 10). Moreover, the layer of cells has covered all surfaces of the channel well (Figure 8, Figure 9 and Figure 10).

To sum up, histological studies have shown that cells in culture subjected to constant flow are able to produce a stable monolayer of cells lining the lumen of the bioprinted vessel. The creation of an internal cell layer prevented leaks, thanks to which the channel continued to remain flow tight over the duration of the experiment. Thanks to this, it will be possible to use the developed technology, e.g., in bioprinted organs intended for transplantation.

## 4. Discussion

Our experimental study provides evidence for the biocompatibility of dECM-based bioink A and its capability to be used in macro vessel formation using a bioprinting technique performed on extrusion bioprinter. Different methods of the fabrication of vascular structures arose recently. The technique based on templating is relatively often in use to produce tubular structures. In this fabrication, a broad range of leachable materials are used, including agarose, gelatin, Pluronic, and various polymers [62,63,64]. The principle of this method is based on embedding the leachable material with a crosslinkable material, and as soon as it jellifies, the core of the leached/liquefied material could be removed. After such a procedure, an empty structure that is reminiscent of a vessel is obtained. Such a strategy was adopted by Ji et al., 2019, who deposited a bioink composed of methacrylate alginate (AlgMa) or methacrylate hyaluronic acid (HAMA) as a crosslinkable material [65]. Afterwards, Pluronic F127 was bioprinted as a leachable, sacrificial ink, which was covered by more layers of crosslinkable bioink. Such a fully photopolymerized construct was than immersed in PBS to dissolve Pluronic and create a perfusable vessel, which afterwards was seeded with HUVEC cells. Although the authors produced channels with different sizes and complexities, the diameters varied from 300 up to 1500 μm, whereas our approach allows us to bioprint a larger vessel with the diameter of 1.6–2.4 mm, which could be implemented in a large-scale scaffold as a main duct for the further formation of multiple branches de novo. The printing procedure, maintaining the appropriate direction of printing individual fibers, guarantees high cell viability and a constant supply of nutrients and gas exchange. The bioprinted flow systems had an elliptical shape. This is related to the extrusion printing technique used. Additionally, it has been shown that the diameter of the nozzle used for bioprinting has a significant impact on the shape of the vessel lumen. When using a larger diameter, the cross-section of the vessel shows various types of irregularities that may affect the adhesion process. When using a narrower nozzle, the inner wall of the vessel is smoother, thanks to which the cells adhere to the biomaterial faster and more permanently. This may be due to a more precise adhesion of the fibers to each other and a tighter printing of subsequent layers. However, it is worth remembering that as cells grow and multiply, the matrix (biomaterial) from which the channel is bioprinted is rebuilt. Such reconstruction may result in smoothing the inner side of the canal [66,67].

The vessel bioprinting presented in this study shares the solution of using leachable Pluronic; however, as presented in experimental variants, it can be substituted by bioink B. The group of Ji et al., 2019, noticed the high viability of HUVEC cells 9 days after they were seeded inside the vessel, and the authors demonstrated their circumferential coverage of a channel [65]. To achieve this, a flipping of a scaffold every 30 min was needed, and the culture took place on an oscillating shaker. The design of the vessel in the following study was made to visualize cells inside, and thus, it was U-shaped in the cross-section. Hence, we flipped it only once so the cells could adhere to walls and the vault of the vessel. Nevertheless, the process of flipping is justified while tending to the total coverage of a channel. Similarly, Kolesky et al., 2014, followed the strategy of using Pluronic in their work; however, the innovation was related with combining different co-cultures, which resulted in different viability [50]. There is a strategy of applying pre-treated scaffolds with fibronectin to facilitate cells adhesion, but in our study, vessels were not covered by any additives before seeding cells, which is a beneficial perspective for the presented dECM-based bioink [68,69].

Bioprinting a vascular system with cells in bioink B appeared to be more efficient than seeding the channel after bioprinting whole organs. Hydrogel B in a temperature of 37 °C becomes a liquid, and a flush of the channel to remove this cell carrier is possible. This strategy is like the procedure of proceeding with leachable materials, such as Pluronic; however, contrary to Pluronic, it takes place in higher temperatures. Thus, it appeared that cells migrated towards solid hydrogel A to settle, and when the media flow was set, those biomechanical forces facilitated cell adhesion [70,71]. It is already provided that perfusion flow has a beneficial effect on endothelial cells, which is reflected with an increase in adhesion and micro-vessel formation under the co-culture conditions [72]. Cell-laden printable materials are not novel in bioengineering. Many researchers up to date successfully incorporated cells in printable ink (adequate viscosity, mechanical properties, crosslinking). Undoubtedly, hydrogels based on alginate and its derivatives constituted a group very frequently used in the last decade. Nevertheless, most of those in vitro studies were made on a micro scale rather than on constructs capable to be used in clinical practice [6,73,74]. Hence, presented in this study, the hydrogel was enriched with GelMa with the concentration of 20% *w*/*v*. GelMa has a great potential as a bioink, but it has been reported that the high concentration (>15% *w*/*v*) needed for maintaining structural fidelity may lead to a decline in cell viability [18,75,76]. Billiet et al., 2014, showed that increasing GelMa concentration with the upper limit of 20% wt% improves the fidelity of biofabricated structures, whereas a lower concentration of GelMa (5 and 10% wt%) leads to the disintegration of printed structure [77]. Thus, the utilization of GelMa in biofabrication ought to be performed with caution to avoid a trade-off between printability and fidelity. Nevertheless, both fibroblasts and endothelial cells employed in this study did not show any behaviour that could suggest that the applied material is inadequate for cell growth. Indeed, a decline in viability before setting up the media flow was reported, but it might be related with the high density of cells suspended in the small volume (50 μL) of medium or bioink B. This hypothesis is supported by the fact that after 24 h of incubation without media flow, the viability decreased below 40% regardless of if the cell carrier was the medium or bioink B. As soon as a fresh supply of media appeared, the viability of cells did not change over the time of the experiment. Unfortunately, no similar research has been conducted in the literature so far, so the authors rely on their own experience in this matter. Many teams do not focus on the technical aspects of bioprinting and try to improve the viability of the cells by modifying the composition of biomaterials, enriching them with various types of additives. Recently, depending on the purpose, different additives are served as a component of hydrogels to increase their specific functionality [78]. Wang et al. [79] successfully embedded nano-hydroxyapatite particles in a hydrogel to promote osteogenesis in bioprinted construct, whereas Kumar et al. [80] employed carboxylated-cellulose nanocrystals (cCNCs) to improve post-printing fidelity. Although the hydrogels presented in this study were not enriched with materials of specific functionality, the dECM was the component that was predicted to facilitate the growth of fibroblasts and endothelial cells [44,45,81,82]. Although the ratio between HUVEC and HDF in the end of the experiments was not determined, the aim for this study was achieved by showing that the bioprinted channel is perfusable and stable for 10 days of the experiment in 37 °C and that cells adhere to the vault and walls of the channel formed with bioink A. Studies on 3D bioprinting and vessel formation are blooming recently [83,84,85,86]. The role of fibroblasts as promotors of extracellular matrix formation could possibly be additionally supported with the dECM of designed hydrogel A, which may facilitate their activity [87,88,89,90,91]. Interestingly, fibroblasts have a wide range of activities depending on the stimulus to which they are responding, and bearing in mind the fact that fibroblasts research community has exploded over the last two decades, presented here, hydrogel A might be a valuable agent for further investigation of their role while exposed to an ECM derived from specific organs [82,92,93,94].

The presented dECM-based bioinks serve an appropriate condition for cell proliferation and adhesion. We claim that the presence of dECM in tested bioinks might be the reason for the behavior of seeded cells. This approach is also confirmed by the literature data describing dECM as a composite that creates the environment surrounding cells, at the same time influencing their functions. In addition to playing an important structural role, the ECM is involved in most basic cell behaviors, from cell proliferation, adhesion, and migration to cell differentiation and cell death [95].

The imaging obtained in IHC staining provides the idea that presented bioink could be employed in various studies, including those related to vasculogenesis as applied cells expressing CD31/PECAM-1. It is likely that bioink A could be utilized in the creation of complex pre-vascularized tissue. A further study concerning the long-time survival of cells and implantation of scaffolds fabricated with bioink A into animal models are necessary to validate the promising outcome of this study. Unfortunately, it is difficult to compare the presented scientific reports with available data because they often do not refer to countering sugars cultured in flow. However, the demonstration of endothelial cell growth from the lumen side of the vessel confirms the effectiveness of the proposed models in achieving an effective vascular system inside 3D constructs.

## 5. Conclusions

In this work, we presented two types of bioinks composed of porcine, pancreatic dECM, and GelMa and HAMA for extrusion-based 3D bioprinting with optimised printing parameters. We showed that the presented bioink, bioink B, can be adopted to studies where cells are expected to be seeded during the bioprinting process within restricted time frame, whereas bioink A is a biocompatible material that forms an adequate condition for cells to adhere and proliferate. Furthermore, bioink A, with the presented fiber arrangement, can be employed for the bioprinting of a macroscale, perfused channel. We demonstrated that applying bioink B during the cell-seeding process on the bioconstruct is unquestionably more effective than using traditional seeding. We conclude that the bioinks presented here have a potential to be used in the biofabrication of 3D vessels and constructs, with the potential for further process of vascularization due to the fact that the applied model cells expressed CD31/PECAM-1 markers.

## Figures and Tables

**Table 1:** Quantity of cells rinsed from channels printed with two techniques (PAR/PER—parallel/perpendicular to media flow) at every incubation time.

Seeding and Fiber Arrangement	Incubation Time Variants			
	2 h	5 h	8 h	24 h
After process—PAR	3.10 × 10^5^	1.93 × 10^5^	2.23 × 10^5^	2.23 × 10^5^
In the process—PAR	3.07 × 10^5^	2.13 × 10^5^	1.87 × 10^5^	3.33 × 10^5^
After process—PER	3.17 × 10^5^	2.60 × 10^5^	2.67 × 10^5^	2.07 × 10^5^
In the process—PER	3.07 × 10^5^	2.67 × 10^5^	2.27 × 10^5^	2.80 × 10^5^

## Data Availability

The raw data supporting the conclusions of this article will be made available by the authors on request.
